# Coinfection of *Pneumocystis jirovecii* and *Aspergillus fumigatus* in the lung: A case report

**DOI:** 10.1097/MD.0000000000050705

**Published:** 2026-09-18

**Authors:** Ming-Hong He, Xiang-Lei Chen, Bao-Tong Feng

**Affiliations:** a Department of Pulmonary and Critical Care Medicine, Yidu Central Hospital of Weifang, Qingzhou, China; b Department of Hematology, Yidu Central Hospital of Weifang, Qingzhou, China.

**Keywords:** *Aspergillus fumigatus*, coinfection, IRIS, *Pneumocystis jirovecii*

## Abstract

**Rationale:**

Coinfection with *Pneumocystis jirovecii* and *Aspergillus fumigatus* in immunocompromised patients carries high mortality. More importantly, paradoxical clinical and radiological responses during treatment remain poorly understood.

**Patient concerns:**

A 66-year-old male with mantle cell lymphoma who had received prolonged corticosteroid therapy after suspected rituximab-associated lung injury presented with progressive pulmonary symptoms.

**Diagnoses:**

Concurrent pulmonary infection with *P jirovecii* and *A fumigatus* was confirmed by bronchoalveolar lavage combined with metagenomic next-generation sequencing.

**Interventions:**

The patient was treated with trimethoprim-sulfamethoxazole and voriconazole.

**Outcomes:**

Clinical symptoms improved markedly; however, chest imaging showed paradoxical progression, possibly reflecting an immune reconstitution inflammatory syndrome-like inflammatory response.

**Lessons:**

bronchoalveolar lavage combined with metagenomic next-generation sequencing enables rapid diagnosis of concurrent opportunistic pulmonary infections. Paradoxical radiographic worsening despite clinical improvement may suggest an immune reconstitution inflammatory syndrome-like inflammatory response, although persistent or progressive infection cannot be excluded.

## 1. Introduction

*Pneumocystis jirovecii* and *Aspergillus fumigatus* are opportunistic fungal pathogens, and simultaneous pulmonary infection caused by these organisms is rare. Early diagnosis is essential for improving outcomes in immunocompromised patients.

## 2. Case presentation

**Figure 1.**
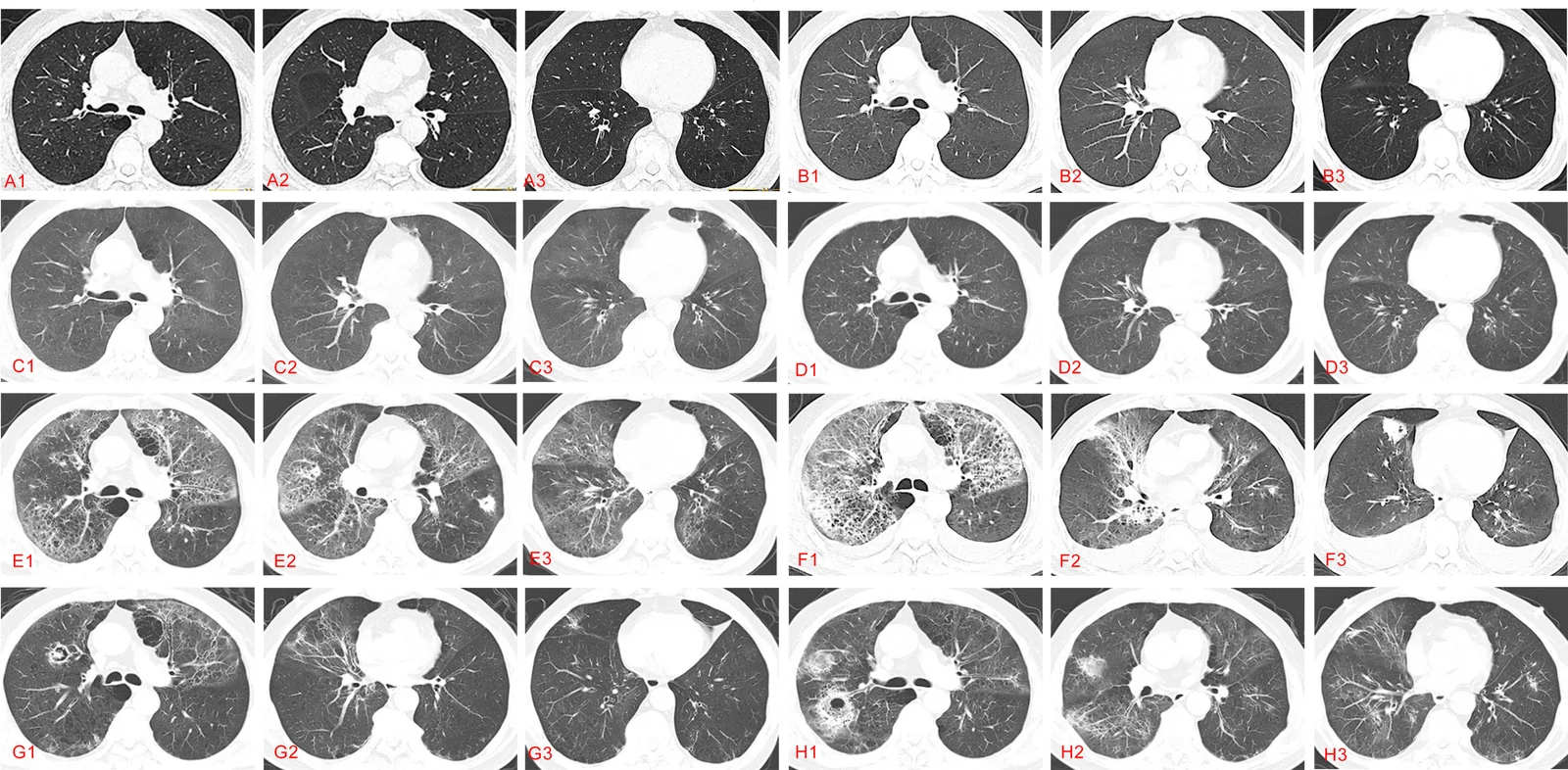
Changes in chest CT imaging. (A1–A3) Bilateral pulmonary emphysema. (B1–B3) Bilateral pulmonary emphysema. (C1–C3) Newly developed multiple large patchy slightly high-density opacities in both lungs. (D1–D3) After methylprednisolone treatment. (E1–E3) Reticular shadows. (F1–F3) Reticular shadows + solid density opacities. (G1–G3) After treatment with TMP/SMX + voriconazole. (H1–H3) Continuing TMP/SMX + voriconazole treatment. CT = computed tomography, TMP/SMX = trimethoprim-sulfamethoxazole.

A 66-year-old man was admitted due to multiple enlarged bilateral cervical lymph nodes. He had a 50-year smoking history (20 cigarettes/day) and underlying pulmonary emphysema (Fig. 1 A1–A3). The patient was diagnosed with stage IIIA mantle cell lymphoma based on positron emission tomography-computed tomography (CT) findings and pathological examination of a left cervical lymph node biopsy.

After 2 cycles of R-CHOP immunochemotherapy (rituximab, cyclophosphamide, pirarubicin, vincristine, and prednisone), a follow-up chest CT showed bilateral pulmonary emphysema without new abnormalities (Fig. 1 B1–B3). One week after completion of the third cycle of R-CHOP immunochemotherapy, the patient developed chest tightness and dyspnea. Chest CT revealed newly developed multiple large patchy areas of slightly increased density in both lungs (Fig. 1 C1–C3). Rituximab-associated lung injury was suspected, and methylprednisolone (40 mg once daily) was administered for 7 days. The patient’s respiratory symptoms improved, and a follow-up chest CT showed partial resolution of the pulmonary lesions (Fig. 1 D1–D3). Oral prednisone (40 mg once daily) was continued after discharge with gradual tapering.

**Figure 2.**
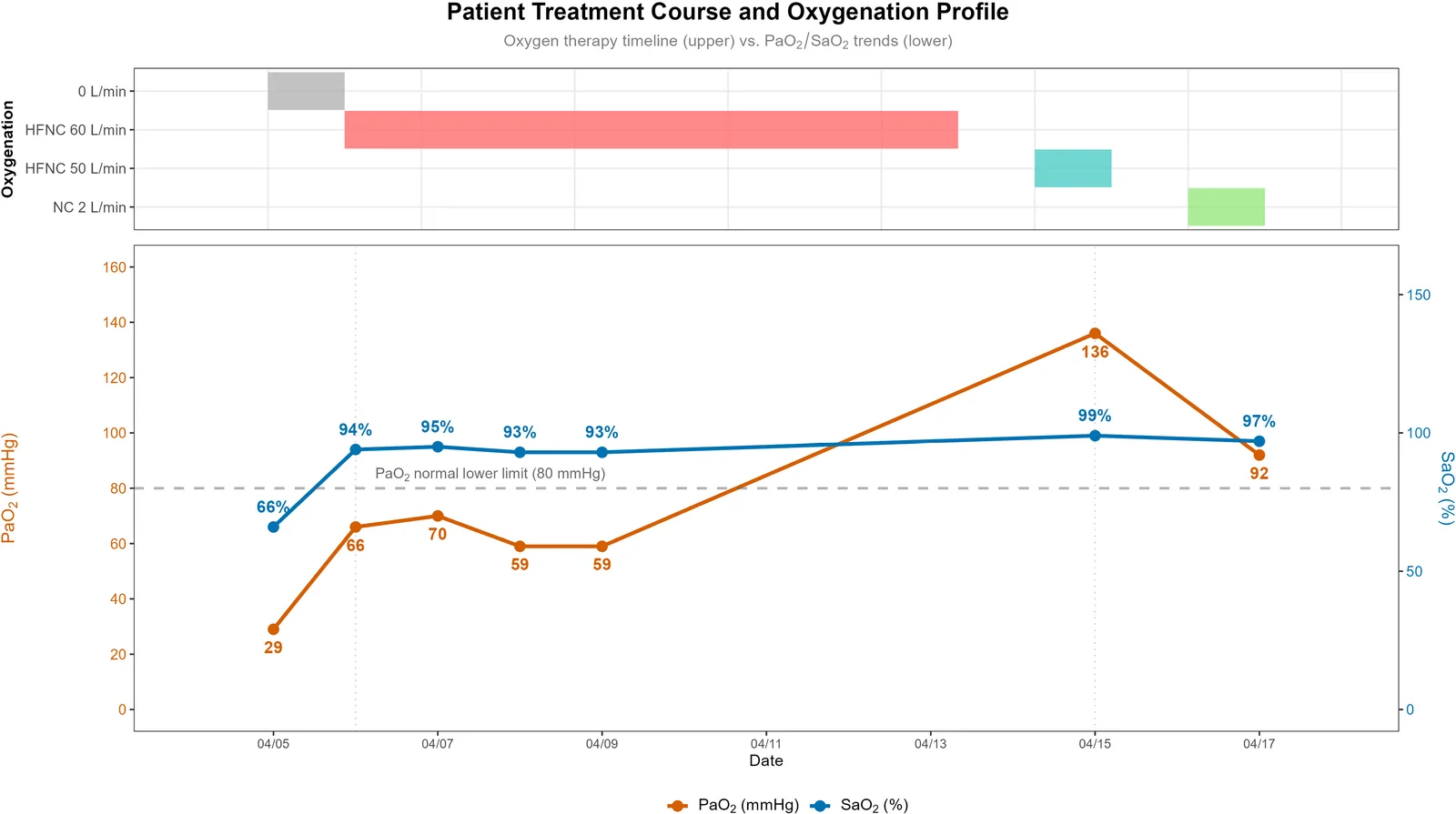
Longitudinal changes in PaO_2_ and SaO_2_ with corresponding oxygen therapy timelines. HFNC = high-flow nasal cannula, NC = nasal cannula, PaO_2_ = partial pressure of oxygen in arterial blood, SaO_2_ = arterial oxygen saturation.

After 21 days of glucocorticoid therapy, the patient developed fever (maximum temperature > 39.0°C), accompanied by chest tightness and dyspnea. Arterial blood gas analysis showed severe hypoxemia, with a partial pressure of oxygen in arterial blood (PaO_2_) of 29 mm Hg and arterial oxygen saturation (SaO_2_) of 66% (Fig. 2). Chest CT demonstrated scattered patchy and reticular opacities in both lungs with focal consolidation and atelectasis (Fig. 1 E1–E3).

Prednisone was continued, and laboratory evaluation showed a serum 1,3-β-D-glucan (BDG) level of 50.5064 pg/mL (reference range < 159 pg/mL), a serum galactomannan (GM) index of 0.13 (reference range < 0.5), and negative aerobic and anaerobic blood cultures. These serum GM and BDG measurements were obtained before initiation of antifungal therapy. No serial serum or BAL fluid (BALF) GM or BDG measurements were performed during the subsequent period of radiological deterioration. High-flow nasal cannula (HFNC) oxygen therapy was initiated at 60 L/min. During HFNC support, PaO_2_ fluctuated between 59 and 70 mm Hg, with SaO_2_ maintained at 93% to 95% (Fig. 2). Empirical treatment with piperacillin/tazobactam (4.5 g every 8 hours) was administered for 5 days; however, no significant clinical improvement was observed.

A follow-up chest CT showed progression of bilateral pulmonary lesions, characterized by increased extent and density of linear and reticular opacities, with patchy consolidation in the right lower lobe (Fig. 1 F1–F3). Therefore, bronchoalveolar lavage (BAL) was performed. BALF was analyzed using metagenomic next-generation sequencing (mNGS). A total of 1,798,701 clean reads were obtained, including 442,063 microbial reads. The analysis detected *P jirovecii* (139 sequences) and *A fumigatus* (630 sequences). After normalization to reads per million (RPM), the abundance of *P jirovecii* was 77.3 RPM and that of *A fumigatus* was 350.2 RPM. Fungal culture and *Aspergillus* antifungal susceptibility testing were not performed on the BALF specimen. Because mNGS detects microbial nucleic acid and does not independently establish organism viability or phenotypic antifungal susceptibility, the detection of *A fumigatus* was interpreted in conjunction with the clinical and radiological findings. BALF immunophenotyping showed no phenotypically abnormal lymphocyte populations. The proportions of CD4^+^ and CD8^+^ T lymphocytes were 28.0% and 72.0%, respectively, with a CD4/CD8 ratio of 0.4. BALF cytokine analysis demonstrated elevated levels of interleukin (IL)-1β (80.91 pg/mL; reference range ≤12.1 pg/mL), IL-6 (84.30 pg/mL; reference range ≤20.0 pg/mL), and IL-8 (1052.87 pg/mL; reference range ≤21.4 pg/mL).

Treatment with trimethoprim-sulfamethoxazole (TMP/SMX, 400/80 mg, 2 tablets 3 times daily) and voriconazole (200 mg every 12 hours) was initiated. After 3 days of therapy, the patient became afebrile, and chest tightness and dyspnea markedly improved. Oxygen support was gradually reduced from HFNC to nasal cannula oxygen (2 L/min), and the patient eventually achieved complete oxygen independence. Follow-up arterial blood gas analysis showed improvement, with PaO_2_ of 92 mm Hg and SaO_2_ of 97% (Fig. 2). Chest CT revealed decreased bilateral linear and reticular opacities, while multiple cavitary lesions were observed. Most cavities had relatively smooth margins; the overall extent of the cavities decreased, although some cavities became enlarged (Fig. 1 G1–G3).

Two weeks later, chest CT showed enlargement of bilateral linear and reticular opacities. Multiple cavitary lesions were observed, including newly developed cavities and enlargement of preexisting cavities. Some cavities contained internal linear or filamentous structures, while most cavity walls remained relatively smooth (Fig. 1 H1–H3).

On May 16, 2026, the patient subsequently refused further chest CT examinations and adjustment of antimicrobial therapy and was lost to follow-up.

The corticosteroid dosing and tapering timeline, together with the available serial peripheral blood neutrophil and lymphocyte counts during the relevant clinical course, are provided in Table S1, Supplemental Digital Content 1, and Table S2, Supplemental Digital Content 2.

## 3. Treatment

Treatment with TMP/SMX (400/80 mg, 2 tablets 3 times daily) and voriconazole (200 mg every 12 hours) was initiated. Voriconazole was administered at twice the maintenance dose for the first 2 doses, after which the maintenance dose was continued. Corticosteroid therapy was continued during antimicrobial treatment (Table S1, Supplemental Digital Content 1). Therapeutic drug monitoring was not performed, and serial renal and hepatic function monitoring specifically for antimicrobial dose adjustment was unavailable.

## 4. Discussion

*P jirovecii* is an important opportunistic fungal pathogen. CD4^+^ T lymphocytes play a central role in host defense against *P jirovecii* pneumonia (PJP). Rituximab-induced depletion of CD20^+^ B lymphocytes reduces antigen-presenting B cells, leading to impaired CD4^+^ T-cell activation and decreased clearance of *P jirovecii*. Rituximab may also reduce anti-*P jirovecii* antibody production, further increasing the risk of PJP.^[1]^ Prolonged glucocorticoid exposure is another major risk factor for PJP.^[1]^ In this patient, rituximab was required for mantle cell lymphoma treatment. During therapy, rituximab-associated lung injury was initially suspected, and the patient subsequently received prolonged corticosteroid therapy. In addition, BALF analysis showed a decreased CD4/CD8 ratio, indicating impaired cellular immunity and increased susceptibility to PJP.

Previous studies have suggested that BALF mNGS with a threshold of >10 RPM may support the diagnosis of PJP.^[2]^ In this case, *P jirovecii* was detected at 77.3 RPM, and the rapid clinical response to TMP/SMX, including resolution of fever and improvement of hypoxemia, supported active infection rather than colonization. Although serum BDG is commonly used for PJP diagnosis, its sensitivity is limited in patients with hematological malignancies.^[3]^ A negative BDG result cannot exclude PJP in this population, as demonstrated in our patient.

Concurrent PJP and pulmonary aspergillosis is uncommon but clinically significant. Approximately 4.5% of immunocompromised patients with PJP may develop concomitant *Aspergillus* infection, particularly those receiving prednisone ≥30 mg/day for more than 4 weeks.^[4]^ BAL combined with mNGS may facilitate early diagnosis and avoid treatment delay.^[5]^ Conventional methods such as GM testing and fungal culture have limitations, including false-negative results and low sensitivity. BALF mNGS has improved diagnostic performance for invasive pulmonary aspergillosis (IPA), and detection above 1 RPM may support IPA diagnosis in immunocompromised patients.^[6]^ In this case, BALF mNGS detected *A fumigatus* at 350.2 RPM. However, because fungal culture and antifungal susceptibility testing were not performed, the mNGS result could not establish organism viability or phenotypic antifungal susceptibility. Furthermore, the low serum GM and BDG levels did not necessarily contradict the BALF mNGS findings. In the context of a predominantly localized pulmonary infection, serum biomarkers may have limited sensitivity, particularly in immunocompromised patients. Therefore, the relatively high abundance of *A fumigatus* detected in BALF by mNGS, despite low serum GM and BDG levels, may represent the limited sensitivity of serum biomarkers for localized pulmonary infection rather than evidence against pulmonary aspergillosis.

Previous studies have demonstrated elevated BALF IL-1β, IL-6, and IL-8 levels in non-human immunodeficiency virus immunocompromised patients with PJP, as well as increased IL-6 and IL-8 levels in hematological patients with IPA.^[7–9]^ Consistent with these observations, our patient showed marked elevation of BALF IL-1β, IL-6, and IL-8, which may reflect a pronounced local inflammatory response. These findings may also be compatible with the exuberant pulmonary inflammatory response postulated in immune reconstitution inflammatory syndrome (IRIS), although they are nonspecific and cannot distinguish IRIS from active infection.

IRIS refers to an excessive inflammatory response triggered by immune recovery against pathogens, resulting in paradoxical clinical deterioration.^[10,11]^ IRIS may occur during effective treatment of both PJP and IPA.^[12,13]^ In this case, fever and respiratory symptoms improved after TMP/SMX and voriconazole therapy, whereas chest CT showed transient radiological progression. This clinical–radiological dissociation raised the possibility of an IRIS-like inflammatory response. However, the mechanism of immune recovery could not be established. Serial CD4^+^ T-cell measurements were not available, and the available peripheral blood data did not demonstrate a clear pattern of immune recovery. Although the neutrophil count increased markedly to 22.67 × 10^9^/L during the period of radiological progression, this increase coincided with high-dose methylprednisolone and was more likely corticosteroid-induced than indicative of immune recovery. The lymphocyte count remained relatively low at 0.84 × 10^9^/L, further arguing against systemic immune reconstitution. Notably, corticosteroid therapy was maintained or intensified during the period of radiological progression rather than substantially reduced. Therefore, the available clinical and hematological data did not establish a definitive temporal relationship between immune recovery and radiological progression.

Serial serum or BALF GM and BDG measurements were not performed during the period of radiological deterioration. The only available serum GM measurement was obtained before antifungal therapy and was negative; therefore, the subsequent trajectory of *Aspergillus*-related biomarkers is unknown, and a single baseline GM measurement cannot distinguish a paradoxical inflammatory response from persistent or progressive pulmonary aspergillosis.

Further radiological follow-up was unavailable because the patient declined additional chest CT examinations and was subsequently lost to follow-up. Consequently, radiological resolution and a self-limited course could not be confirmed. Persistent or progressive infection, delayed radiological resolution, and inflammatory changes associated with tissue repair therefore remain alternative explanations. Accordingly, the radiological progression in this case may represent an IRIS-like inflammatory response, although persistent or progressive infection and other alternative explanations cannot be excluded.

The development and progression of cavitary lesions also warrant consideration of alternative explanations. PJP typically presents with bilateral ground-glass opacities,^[14]^ whereas cavitation is uncommon. In contrast, pulmonary aspergillosis is a well-recognized cause of cavitary lesions due to tissue necrosis, making *A fumigatus* a plausible contributor in our patient. In addition, the patient’s emphysema and 50-year smoking history may have contributed to preexisting structural lung abnormalities that influenced the development or morphology of the cavities. Although paradoxical radiological worsening has been described in IRIS, cavitation is not a characteristic imaging feature. Therefore, the observed cavitary evolution was likely multifactorial and should not be attributed to a single mechanism. Because our patient declined further treatment and follow-up, the effectiveness of subsequent management could not be evaluated. Nevertheless, this case highlights the importance of establishing a structured posttreatment monitoring and prevention strategy, including reassessment of the need for secondary PJP prophylaxis, continuation of antifungal therapy or secondary antifungal prophylaxis when clinically indicated, and serial clinical, radiological, and laboratory monitoring in patients with opportunistic pulmonary infection.

## 5. Conclusion

Lymphoma patients receiving rituximab and prolonged glucocorticoid therapy are at increased risk of simultaneous pulmonary infection with *P jirovecii* and *A fumigatus*. For suspected cases, comprehensive evaluation using chest CT, BAL, and BALF mNGS may enable early diagnosis and appropriate treatment, thereby improving clinical outcomes. Clinical improvement accompanied by paradoxical radiological worsening may raise the possibility of an IRIS-like inflammatory response; however, persistent or progressive infection should be carefully excluded, particularly when serial biomarker monitoring and long-term follow-up are unavailable.

## Acknowledgments

The authors used DeepSeek solely for English language editing and translation. The tool was not involved in the study conception, data analysis, or the generation of scientific conclusions. The authors take full responsibility for the final content.

## Author contributions

**Conceptualization:** Ming-Hong He, Xiang-Lei Chen.

**Data curation:** Ming-Hong He, Xiang-Lei Chen.

**Formal analysis:** Bao-Tong Feng.

**Writing – original draft:** Xiang-Lei Chen.

**Writing – review & editing:** Bao-Tong Feng.




